# HIV Incidence, Recent HIV Infection, and Associated Factors, Kenya, 2007–2018

**DOI:** 10.1089/aid.2022.0054

**Published:** 2023-02-08

**Authors:** Peter Wesley Young, Paul Musingila, Leonard Kingwara, Andrew C. Voetsch, Emily Zielinski-Gutierrez, Marc Bulterys, Andrea A. Kim, Megan A. Bronson, Bharat S. Parekh, Trudy Dobbs, Hetal Patel, Giles Reid, Thomas Achia, Alfred Keter, Samuel Mwalili, Francis M. Ogollah, Raphael Ondondo, Herbert Longwe, Duncan Chege, Nancy Bowen, Mamo Umuro, Catherine Ngugi, Jessica Justman, Peter Cherutich, Kevin M. De Cock

**Affiliations:** ^1^ Division of Global HIV & TB, U.S. Centers for Disease Control and Prevention, Maputo, Mozambique.; ^2^ Division of Global HIV & TB, U.S. Centers for Disease Control and Prevention, Nairobi, Kenya.; ^3^ National AIDS & STI Control Programme, Ministry of Health, Nairobi, Kenya.; ^4^ Division of Global HIV & TB, U.S. Centers for Disease Control and Prevention, Atlanta, Georgia, USA.; ^5^ Central America Regional Office, U.S. Centers for Disease Control and Prevention, Guatemala City, Guatemala.; ^6^ Survey Unit, ICAP at Columbia University, New York, New York, USA.; ^7^ Department of Statistics and Actuarial Sciences, Jomo Kenyatta University of Agriculture and Technology, Juja, Kenya.; ^8^ National Public Health Laboratory, Ministry of Health, Nairobi, Kenya.; ^9^ Ministry of Health, Nairobi, Kenya.

**Keywords:** HIV surveillance, HIV incidence estimation, antiretroviral treatment, tests for recent infection, population surveys, Kenya

## Abstract

Nationally representative surveys provide an opportunity to assess trends in recent human immunodeficiency virus (HIV) infection based on assays for recent HIV infection. We assessed HIV incidence in Kenya in 2018 and trends in recent HIV infection among adolescents and adults in Kenya using nationally representative household surveys conducted in 2007, 2012, and 2018. To assess trends, we defined a recent HIV infection testing algorithm (RITA) that classified as recently infected (<12 months) those HIV-positive participants that were recent on the HIV-1 limiting antigen (LAg)-avidity assay without evidence of antiretroviral use. We assessed factors associated with recent and long-term (≥12 months) HIV infection versus no infection using a multinomial logit model while accounting for complex survey design. Of 1,523 HIV-positive participants in 2018, 11 were classified as recent. Annual HIV incidence was 0.14% in 2018 [95% confidence interval (CI) 0.057–0.23], representing 35,900 (95% CI 16,300–55,600) new infections per year in Kenya among persons aged 15–64 years. The percentage of HIV infections that were determined to be recent was similar in 2007 and 2012 but fell significantly from 2012 to 2018 [adjusted odds ratio (aOR) = 0.31, *p* < .001]. Compared to no HIV infection, being aged 25–34 versus 35–64 years (aOR = 4.2, 95% CI 1.4–13), having more lifetime sex partners (aOR = 5.2, 95% CI 1.6–17 for 2–3 partners and aOR = 8.6, 95% CI 2.8–26 for ≥4 partners vs. 0–1 partners), and never having tested for HIV (aOR = 4.1, 95% CI 1.5–11) were independently associated with recent HIV infection. Although HIV remains a public health priority in Kenya, HIV incidence estimates and trends in recent HIV infection support a significant decrease in new HIV infections from 2012 to 2018, a period of rapid expansion in HIV diagnosis, prevention, and treatment.

## Introduction

There were 1.4 million adolescents and adults aged 15 years and older estimated to be living with human immunodeficiency virus (HIV) in Kenya in 2021.^1^ The country has rapidly expanded access to HIV testing, resulting in a 4.4-fold increase in self-reported knowledge of HIV-positive status among adolescents and adults aged 15–64 years from 2007 to 2018.^2,3^ Coverage of antiretroviral treatment (ART) among all people living with HIV has increased 2.6-fold over the same period, and the percentage of males that are uncircumcised has fallen by nearly half, from 15.0% to 8.3%.^2,3^

Tracking the incidence of HIV infection over time can provide the ideal evidence of control of the HIV epidemic within a population; however, measuring it accurately presents challenges. Available methods include prospective cohorts,^4,5^ mathematical models,^6–9^ and tests for recent HIV infection applied in cross-sectional surveys.^10^ In many settings, cross-sectional surveys may be more practical than national prospective cohorts, which are complex and suffer challenges with external validity, especially over extended time frames.^4^ Tests for recent HIV infection based on antibody maturation, such as the HIV-1 limiting antigen (LAg)-avidity assay, have been used extensively to estimate HIV incidence.^11,12^ When used in a recent HIV infection testing algorithm (RITA) that combines LAg-avidity results with supplementary laboratory and clinical information, they can perform well compared to traditional cohort estimates.^13^

Prior reports have estimated HIV incidence among adolescents and adults aged 15–64 years in Kenya at 0.5%–1.0% from the 2007 and 2012 Kenya AIDS Indicator Surveys (KAIS) using both LAg-avidity and model-based approaches.^8,14,15^ The Joint United Nations Programme on HIV/AIDS (UNAIDS) estimated new HIV infections to have fallen by 48% in Kenya from 2010 to 2020, demonstrating significant progress, but below the 75% reduction target.^1,16^

Although large community-randomized trials have found that increasing ART coverage through universal test and treat leads to reduced population viremia and reduced HIV transmission, continuing scale-up of ART may be insufficient to eliminate HIV as a public health threat.^17^ Identifying individual-level factors associated with recent HIV infection may be useful for targeting HIV prevention among those at risk. Previous studies have identified sexually transmitted infections (STIs), sexual behaviors, lack of male circumcision, and marital status as factors associated with recent HIV infection in Kenya.^18,19^ In this report, we use three national population-based surveys to describe HIV incidence in 2018, factors associated with recent (<12 months) and long-term HIV infection in 2012 and 2018, and trends in the percentage of HIV infections that are recent in Kenya from 2007 to 2018.

## Methods

### Survey sample and population

The KAIS 2007, KAIS 2012, and the Kenya population-based HIV Impact Assessment (KENPHIA) 2018 were national, population-based surveys that used multistage sample designs based on successive master sampling frames developed by the Kenya National Bureau of Statistics. All three surveys were designed to produce national, urban, and rural estimates. In addition, KAIS 2007 was designed to produce representative estimates for the eight former provinces of Kenya, while KAIS 2012 was designed to produce representative estimates for nine subnational domains that excluded the less populated North Eastern region due to security issues. KENPHIA was designed to produce representative estimates for all 47 counties created under the 2010 Constitution of Kenya. The original survey methods are described in detail elsewhere.^2,3,20^

### Survey procedures

All three surveys included behavioral questionnaires and testing for HIV infection, followed by both HIV ribonucleic acid (RNA) testing for viral load estimation and the LAg-avidity assay for recent HIV-1 infection among those with a positive HIV test result. Due to issues with the quality of stored specimens from KAIS 2007 that affected viral load testing, viral load results from 2007 were unavailable. The survey questionnaires included questions on ART use for persons who self-identified as HIV-positive, and biomarker testing for the qualitative detection of selected antiretroviral (ARV) medications was conducted on samples from all HIV-infected respondents, regardless of self-identification of positive status, as described below.

Survey participants provided written informed consent before participation and were provided access to their HIV testing results. In the case of KAIS 2007 and 2012, after completing other survey procedures, participants were offered rapid HIV testing with voluntary counseling and testing with return of results via a nearby health facility (2007) or with rapid testing in the home with immediate return of results after survey procedures were concluded (2012). Blood specimens collected during the survey were then used to estimate HIV prevalence using enzyme-linked immunosorbent assay (ELISA)-based tests, followed by additional confirmatory testing in the central laboratory.

In KENPHIA, participants were offered rapid HIV testing in the home with immediate return of results as part of the survey. After laboratory confirmation with Geenius HIV-1/2 supplemental assay (Bio-Rad, Marnes-la-Coquette, France), these results were used to estimate HIV prevalence. Further details on the HIV testing algorithms used for HIV prevalence estimation are provided in the respective survey reports.^2,14,21^

### Laboratory methods

The LAg-avidity assay was used to detect recent HIV infection at the central laboratory from serum samples in KAIS 2007 and dried blood spots (DBS) in KAIS 2012, while in KENPHIA, plasma was used with DBS available as a backup in case plasma was not available or depleted by preceding tests. LAg-avidity kits from Sedia Biosciences (Portland, OR) were used for testing plasma or serum samples, while DBS samples were tested using kits from Maxim Biomedical (Rockville, MD). KAIS 2012 used DBS to measure HIV RNA levels using the Abbott M2000 Real-Time HIV-1 Assay (Abbott Laboratories, Abbott Park, IL). For KENPHIA, RNA measurement was performed on plasma using the automated Roche COBAS AmpliPrep/COBAS TaqMan (CAP/CTM) HIV-1 RNA quantitation test (Roche Molecular Systems, Inc., Pleasanton, CA) or on DBS with the Roche CAP/CTM Free Virus Elution (FVE) Protocol.

DBS were tested to qualitatively detect ARVs in blood using tandem mass spectrometry liquid chromatography in all three surveys.^22^ The panel of ARVs evolved over time, reflecting changes in treatment guidelines: the ARVs tested for in the 2007 and 2012 surveys included nevirapine, efavirenz, lamivudine, and lopinavir, while in the 2018 survey, efavirenz, nevirapine, and atazanavir were included. Due to varying pharmacokinetics, efavirenz and nevirapine can be detected for up to 28 and 9 days, respectively, while the other included drugs can be detected for 1–3 days postingestion on DBS before their levels fall below the assay limit of detection due to their shorter half-lives.^23^ ARV testing was conducted at the University of Cape Town in 2014 for KAIS 2007 and 2012 and in 2019 for KENPHIA.

### Recency estimation

Due to differences in available biomarkers for each survey, two different RITAs were used to classify participants as having recent HIV infection. For both algorithms, ART use was defined as self-reported ART use or detection of ARVs in blood, while lack of ART use was defined as having neither. In the 2012 and 2018 surveys, participants were classified as follows: those with HIV-positive specimens that were recent according to LAg-avidity and were not virally suppressed (viral load ≥1,000 copies/mL [c/mL] of plasma) nor on ART were considered recent; others with an HIV-positive survey specimen were considered long-term. The LAg-avidity cutoffs for recent infection were median normalized optical density (ODn) ≤1.5, except in KENPHIA where ODn ≤1.0 was used for DBS specimens due to recalibration. This algorithm is referred to hereafter as the “full RITA.”

**FIG. 1.**
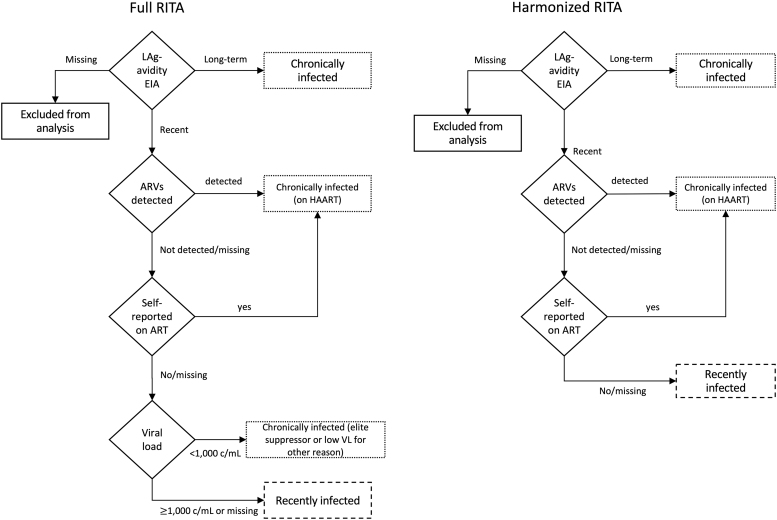
Recent HIV infection testing algorithms. ART, antiretroviral treatment; ARV, antiretroviral; c/mL, copies per mL; EIA, enzyme-linked immunosorbent assay; HIV, human immunodeficiency virus; LAg, limiting antigen; RITA, recent HIV infection testing algorithm; VL, viral load.

**FIG. 2.**
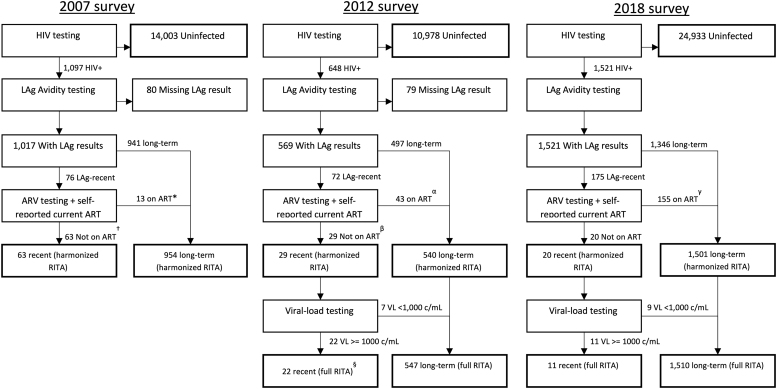
Recent HIV infection testing status for specimens according to harmonized and full RITA, Kenya, 2007–2018. All numbers are unweighted and exclude North Eastern region. *Includes four specimens with missing ARV test result (self-reported not on ART). ^†^Includes 22 specimens with missing ARV test result (self-reported not on ART). §Includes one specimen with missing VL result. ^α^Includes five specimens with missing ARV test (self-reported on ART). ^β^Includes six specimens with missing ARV test (self-reported not on ART). ^γ^Includes two specimens with positive ARV test result but missing self-reported ART status. Harmonized RITA is based on LAg and ART (self-report and biomarker), full RITA is based on LAg, ART (self-report and biomarker) and VL ≥1,000 c/mL. ART, antiretroviral treatment; RITA, recent HIV infection testing algorithm.

The available biomarkers and survey questions were assessed across the three surveys to define a RITA that could be applied consistently over time. As self-reported current ART use and ARV testing were included in all three surveys, we defined a “harmonized RITA” which classified participants as “recent” if their HIV-positive specimens were recent on LAg-avidity and not on ART; others with an HIV-positive survey specimen were considered long-term. For both RITAs, participants with no LAg-avidity outcome were excluded from analysis, while those with a missing result for the other biomarkers were classified according to the remaining biomarkers (Figs. 1 and 2). As a sensitivity analysis, we compared the percentage of HIV-positive respondents classified as recent using the full versus harmonized RITAs for the 2012 and 2018 surveys (the years when it was possible to compute both RITAs).

Factors identified *a priori* were tabulated versus HIV status (recent, long-term, or uninfected). Those with preliminary evidence of association (*p* < .1) in bivariate analysis were included in multinomial logit models to identify factors independently associated with recent or long-term HIV infection versus noninfection. This analysis was limited to the 2012 and 2018 surveys to facilitate the use of the more robust full RITA with reduced risk of individual-level misclassification and provide a more relevant time period for applicability of results. Those classified as recent on either RITA were considered to have been infected approximately within the last year, while those classified as long-term were considered to have been infected >1 year before the survey.

### Estimation of HIV incidence and new HIV infections

For purposes of estimating HIV incidence using the full RITA, we use the incidence estimator proposed by Kassanjee et al^24^ with false-recency proportion set to 0, mean duration of recent infection set to 130 days and the maximum time for which recency was evaluated (big T) set to 1 year (365 days) as per Centers for Disease Control and Prevention (CDC) recommendations.^13^ Point estimates and confidence intervals (CIs) for incidence were estimated using a SAS macro developed for the population-based HIV Impact Assessment (PHIA) surveys using the unmodified replicate KENPHIA survey weights for jackknife variance estimation. Parametric annual HIV incidence estimates were combined with the projected population size provided by the Kenya National Bureau of Statistics to estimate newly infected persons by year, age, and sex in 2018.

### Statistical methods

Datasets were harmonized and combined to reduce the possibility of spurious findings due to methodological differences between surveys, as described previously.^25^ In brief, the North Eastern region was excluded from the 2007 and 2018 survey datasets, and weights for the 2018 survey were revised to use a nonresponse adjustment based on region, age, and sex consistent with the 2012 survey. We performed a sensitivity analysis to estimate the impact of these changes on trends in HIV recency. Differences in the percentage of infections that were recent in each survey were assessed for statistical significance using logistic regression with survey year treated as a nominal variable while adjusting for age and sex.

Multinomial logistic regression was used to assess associations between explanatory variables and recent or long-term versus noninfection, while adjusting for potential confounders such as survey year, sex, age, and province of residence. We conducted a sensitivity analysis to assess the impact of model selection on the findings. Sampling from each survey was assumed to be independent, and all analyses accounted for the complex survey designs. Sample counts and response rates are unweighted. An additional exploratory analysis of the association of HIV testing history and recency of HIV infection was done by plotting the time since most recent HIV test versus recent infection status. All analyses were conducted in SAS 9.4, statistical tests were conducted at 5% level of significance, and 95% CIs were reported for all estimates, which were rounded to two significant digits of precision, except for population counts that were rounded to the nearest 100.

### Ethical considerations

All three survey protocols were reviewed and approved by the Kenya Medical Research Institute Ethics Review Committee and the U.S. CDC Institutional Review Board (IRB). The KAIS 2007 and 2012 were also approved by the Committee on Human Research of the University of California, San Francisco, and KENHPIA was approved by the Columbia University Medical Center IRB.

## Results

During KENPHIA, 30,384 participants aged 15–64 years consented to a face-to-face interview, and of these, 27,745 (91%) consented to HIV testing. Of those tested for HIV, 1,523 (5.5%) were HIV-positive, and of these, 2 resided in the North Eastern region and hence were excluded from comparisons between surveys. Of the remaining 1,521 participants, 175 (12%) had samples with an ODn ≤1.5 (or 1.0 for 32 specimens tested on DBS using the Maxim kit), of which 20 had no evidence of ARV use, and of these 20 participants, 11 had viral load ≥1,000 c/mL (Fig. 2).

Annual HIV-1 incidence was 0.14% (95% CI 0.057–0.23) among adolescents and adults aged 15–64 years in 2018 using the full RITA. This incidence estimate is equivalent to 35,900 (95% CI 16,300–55,600) annual new HIV infections among Kenyans aged 15–64 years in 2018. HIV incidence estimates did not differ significantly between males and females nor by age; however, the limited number of recent infections among subgroups by age and sex did not allow for detailed subgroup analyses (Table 1) nor comparisons of HIV incidence estimates from earlier years.

**Table 1.** HIV Incidence and New Infections, by Age Group and Sex, Kenya Population-Based HIV Impact Assessment 2018

	*N* recent (unweighted)	% Annual incidence (95% CI)	Annual new infections (95% CI)
Age (years)			
15–24	1	0.071 (0.00–0.16)	6,600 (0–15,400)
25–34	4	0.22 (0.00–0.46)	15,600 (2,500–28,800)
35–44	4	0.16 (0.00–0.35)	7,200 (0–16,200)
45–64	2	0.15 (0.00–0.37)	6,500 (0–16,300)
Sex			
Male	4	0.13 (0.020–0.24)	16,800 (2,500–31,100)
Female	7	0.15 (0.013–0.29)	19,100 (1,600–36,700)
Total	11	0.14 (0.057–0.23)	35,900 (16,300–55,600)
Total, 15–49 years	10	0.15 (0.058–0.24)	34,200 (14,600–53,800)

Uses RITA based on LAg, viral load ≥1,000 c/mL and ART use, MDRI = 130 days, time cutoff (big T) = 365 days, and PFR = 0. Estimated number of annual new infections is estimated by multiplying the incidence rate (and confidence limits) by the population for the respective age/sex band for Kenya in 2018 according to census projections. Percentages rounded to two significant digits and population counts rounded to nearest 100.

ART, antiretroviral treatment; CI, confidence interval; c/mL, copies/mL; HIV, human immunodeficiency virus; LAg, limiting antigen; MDRI, mean duration of recent infection; PFR, proportion false-recent; RITA, recent HIV infection testing algorithm.

The percentages of all persons with HIV who tested recent using a harmonized RITA are shown in Table 2. In KAIS 2007, 63 (6.3%) of 1,017 HIV-positive participants with LAg-avidity results were classified as recent. In KAIS 2012, 29 (5.6%) of 569 participants were classified as recent, while in KENPHIA, 20 (1.7%) of 1,521 participants were classified as recent. In 2007, 22 LAg-recent participants were missing ARV biomarker results and were classified based on self-reported ART status, while 6 such cases occurred in 2012 (there were no missing ARV biomarker results in 2018). Further details on how cases were classified with respect to ART status are provided in Supplementary Table S1.

**Table 2.** Percentage of Recent Infections Among HIV Positives by Year and by Recent Infection Testing Algorithm, Kenya, 2007–2018

		Male	Female	15–24 Years	25–34 Years	35–44 Years	45–64 Years	Total
Harmonized RITA								
2007 (*N* = 1,017)	*n* (%)	26 (7.7)	37 (5.7)	18 (11)	26 (7.7)	9 (3.0)	10 (4.1)	63 (6.3)
	95% CI	3.6–12	3.4–7.9	5.1–16	4.2–11	0.60–5.5	1.1–7.2	4.2–8.4
2012 (*N* = 569)	*n* (%)	8 (5.9)	21 (5.4)	9 (15)	13 (8.4)	5 (3.1)	2 (1.4)	29 (5.6)
	95% CI	0.37–11	2.9–8.0	3.7–27	3.5–13	0.19–6.0	0–3.3	3.0–8.2
	aOR (*p*-value) vs. 2007	0.80 (0.70)	1.2 (0.64)	1.4 (0.49)	1.1 (0.90)	1.0 (0.97)	0.29 (0.15)	1.00 (1.0)
2018 (*N* = 1,521)	*n* (%)	7 (2.5)	13 (1.3)	5 (6.2)	5 (1.8)	5 (0.82)	5 (1.1)	20 (1.7)
	95% CI	0.34–4.6	0.42–2.1	0–13	0.031–3.6	0–1.7	0–2.3	0.75–2.6
	aOR (*p*-value) vs. 2007	**0.35 (0.047)**	**0.27 (0.0014)**	0.51 (0.26)	**0.23 (0.0084)**	0.27 (0.052)	**0.24 (0.032)**	**0.31 (<0.001)**
	aOR (*p*-value) vs. 2012	0.45 (0.21)	**0.23 (<0.001)**	0.36 (0.15)	**0.22 (0.0096)**	0.26 (0.065)	0.84 (0.85)	**0.31 (0.0018)**
Full RITA								
2012 (*N* = 569)	*n* (%)	6 (5.0)	16 (3.6)	7 (11)	11 (7.1)	4 (2.4)	0	22 (4.1)
	95% CI	0–10	1.6–5.6	0.59–21	2.5–12	0–5.0	—	1.7–6.5
2018 (*N* = 1,521)	*n* (%)	4 (1.4)	7 (0.73)	1 (2.2)	4 (1.2)	4 (0.64)	2 (0.65)	11 (0.95)
	95% CI	0–3.0	0.069–1.4	0–6.5	0–2.6	0–1.4	0–1.7	0.27–1.6
	aOR (*p*-value) vs. 2012	0.33 (0.16)	**0.20 (0.0036)**	0.18 (0.13)	**0.17 (0.0084)**	0.26 (0.11)	— (—)	**0.26 (0.0032)**

RITA, *n*/*N* is number recent over total HIV+ tested for recency (unweighted), CI, aOR, controlling for sex and age group. All estimates adjusted for survey designs. Harmonized RITA is based on LAg-avidity and antiretroviral treatment (self-report and biomarker), full RITA is based on LAg-avidity assay, antiretroviral treatment (self-report and biomarker) and viral load ≥1,000 c/mL. Items in bold are significant (*p* < .05). Percentages, *p*-values, and odds ratios rounded to two significant digits.

aOR, adjusted odds ratio.

Adjusting for sex and age group, the percentage recent decreased significantly from 2007 to 2018 [adjusted odds ratio (aOR) = 0.31, *p* < .001] and from 2012 to 2018 (aOR = 0.31, *p* = .0018), although not from 2007 to 2012 (aOR = 1.0, *p* = 1.0). Significant decreases were seen for males (aOR = 0.35, *p* = .047) and females (aOR = 0.27, *p* = .0014), and for 25- to 34- (aOR = 0.23, *p* = .0084), and 45- to 64- (aOR = 0.24, *p* = .032)-year-olds from 2007 to 2018. From 2012 to 2018, significant differences were seen for females (aOR = 0.23, *p* < .001) and 25- to 34-year-olds (aOR = 0.22, *p* = .0096). Using the full RITA, which was only possible for 2012 and 2018, seven and nine fewer specimens were classified as recent in 2012 and 2018, respectively.

The reduction in percentage recent from 2012 to 2018 was also significant using this model (aOR = 0.26, *p* = .0032), including for females (aOR = 0.20, *p* = .0036) and 25- to 34-year-olds (aOR = 0.17, *p* = .0084), but not for other studied subgroups (Table 2). A sensitivity analysis using the original survey weights and including North Eastern in 2007 and 2018 surveys did not alter the findings (Supplementary Table S2).

After pooling the weighted 2012 and 2018 survey results, recent infections were evenly divided by sex, while 65% of long-term infections had occurred in females (Table 3). While 26% of recent infections occurred in the 15- to 24-year-olds and 5.1% among 45- to 64-year-olds, 9.3% of long-term infections were found in 15- to 24-year-olds and 31% among 45- to 64-year-olds, respectively. The majority of both recent (64%) and long-term (58%) infections were among those who were married or living together with a partner. Almost 9 out of 10 recent infections were among those reporting 2 or more lifetime sex partners, whereas roughly half (56%) of the uninfected were in these categories.

**Table 3.** Pooled Analysis of Demographic, Clinical, and Behavioral Characteristics of Adults in Kenya by HIV Infection Status, in 2012 and 2018, Combined (*N* = 38,001)

Characteristic	Recent HIV infection*^a^* (*N* = 33)			Long-term HIV infection*^a^* (*N* = 2,057)			HIV-uninfected (*N* = 35,911)		
	N^b^	Col %	95% CI	N^b^	Col %	95% CI	N^b^	Col %	95% CI
Year									
2012	22	78	61–94	547	44	40–48	10,978	46	44–48
2018	11	22	6.0–39	1,510	56	52–60	24,933	54	52–56
Sex									
Male	10	47	21–73	583	35	32–38	15,427	50	49–51
Female	23	53	27–79	1,474	65	62–68	20,484	50	49–51
Residency									
Urban	12	37	9.4–64	837	41	37–45	12,864	38	36–40
Rural	21	63	36–91	1,220	59	55–63	23,047	62	60–64
Province									
Nairobi	2	4.1	0–11	107	9.7	7.3–12	2,400	12	11–13
Central	1	3.6	0–11	136	7.2	5.8–8.7	3,847	13	12–14
Coast	4	7.4	0–16	157	7.3	5.7–8.9	4,098	9.6	8.5–11
Eastern	5	25	0–55	255	9.6	7.4–12	7,235	14	13–15
Nyanza	9	21	5.7–36	834	37	33–41	4,905	13	11–14
Rift Valley	6	20	3.1–36	371	20	17–23	9,722	28	27–30
Western	6	20	3.0–37	197	9.2	7.0–11	3,704	10	9.5–11
Age group (years)									
15–24	8	26	10–42	191	9.3	7.8–11	11,905	34	33–34
25–34	15	50	33–67	617	29	27–32	9,594	27	26–28
35–44	8	19	2.5–35	600	30	28–33	6,725	19	18–19
45–64	2	5.1	0–13	649	31	28–33	7,687	20	20–21
Education									
No education	2	0.34	0–0.87	158	6.7	5.3–8.1	3,273	6.6	5.7–7.6
Primary	25	85	71–99	1,348	65	62–68	19,055	53	52–54
≥Secondary	6	15	1.0–28	551	29	26–31	13,581	40	39–42
Marital status									
Never married	5	13	0.44–26	235	10	8.6–12	11,201	32	32–33
Separated/Divorced/Widowed	7	23	5.1–41	657	32	29–35	3,852	9.8	9.3–10
Married/Living together	21	64	42–85	1,164	58	55–61	20,812	58	57–59
Wealth index									
I (lowest)	7	19	3.2–35	437	17	15–20	8,544	19	18–21
II	10	26	7.1–44	502	24	21–27	8,114	21	20–22
III	5	14	0.23–29	490	23	20–26	7,536	20	19–21
IV	5	13	0–26	411	21	18–24	6,570	20	18–21
V (highest)	6	28	0–57	216	15	13–18	5,144	20	18–22
Lifetime sex partners									
0–1	6	9.5	0–19	427	19	17–22	15,772	41	40–41
2–3	12	40	24–57	793	36	33–39	10,776	30	29–31
≥4	12	46	29–63	735	40	38–43	7,830	26	25–27
Unknown/Missing	3	4.4	0–9.9	102	4.2	3.0–5.4	1,533	3.3	2.9–3.6
Genital discharge in last 12 months									
Yes	4	7.7	0–19	215	9.2	7.8–11	1,970	5.1	4.8–5.5
No	29	92	81–100	1,831	91	89–92	32,300	95	95–95
Genital ulcer/sore in last 12 months									
Yes	3	3.9	0–8.8	168	7.8	6.4–9.3	1,198	3.1	2.9–3.4
No	30	96	91–100	1,868	92	91–94	33,002	97	97–97
Used condom at last sex in last 12 months									
Yes	4	7	0–16	626	31	28–34	3,680	12	11–12
No	21	80	66–94	728	36	34–39	20,050	57	56–58
Not sexually active	4	7.5	0–16	551	26	24–29	10,033	27	26–28
Unknown/Missing	4	5.5	0–12	152	6.4	5.1–7.7	2,148	5	4.6–5.4
Circumcision status (males only)^c^									
Circumcised	8	83	59–100	390	68	62–73	14,073	92	91–93
Not circumcised	2	17	0–41	192	32	27–38	1,336	7.8	7.0–8.5
Ever tested for HIV									
Yes	25	54	28–81	1,923	93	91–94	27,282	75	74–76
No	8	46	19–72	132	7.3	5.8–8.9	8,543	25	24–26

^a^ Recent and long-term HIV infection according to full RITA based on LAg-avidity assay, antiretroviral treatment (self-report and biomarker) and viral load ≥1,000 c/mL.

^b^ *N* are unweighted.

^c^ One participant excluded in 2012 and nine excluded in 2018 due to missing response. Percentages rounded to two significant digits.

In multivariable analysis, the odds of recent infection versus no infection were greater among those surveyed in 2012 (aOR = 3.2, 95% CI 1.2–8.5), aged 25–34 (aOR = 4.2, 95% CI 1.4–13) versus 35–64 years old, having either 2–3 (aOR = 5.2, 95% CI 1.6–17) or ≥4 (aOR = 8.6, 95% CI 2.8–26) versus 0–1 lifetime sex partners, or having never been tested for HIV (aOR = 4.1, 95% CI 1.5–11) (Table 4). Although prior HIV testing and having no (vs. a recent) HIV infection were associated in the pooled regression analysis, this no longer appeared to be the case by 2018. Further, there was no consistent gradient in the proportion recent by time since last HIV test, among those who had ever been tested (Supplementary Fig. S1 and Supplementary Table S3).

**Table 4.** Multivariable Analysis of Factors Associated with Recent and Long-Term HIV Infection in Multinomial Logistic Regression, Kenya 2012 and 2018 Combined (*n* = 38,001)

Characteristic	Recent infection compared to HIV-uninfected		Long-term infection compared to HIV-uninfected	
	aOR	95% CI	aOR	95% CI
Year				
2012	**3.2**	**1.2–8.5**	0.92	0.79–1.1
2018 (Ref.)				
Sex				
Male (Ref.)				
Female	2.4	0.77–7.5	**3.1**	**2.6–3.7**
Province				
Nyanza/Western	1.9	0.76–4.8	**2.3**	**1.9–2.6**
Other (Ref.)				
Age group (years)				
15–24	3.1	0.76–13	**0.17**	**0.13–0.20**
25–34	**4.2**	**1.4–13**	**0.64**	**0.56–0.74**
35–64 (Ref.)				
Education				
No education/Primary (Ref.)				
≥Primary	2.8	0.88–9.1	**1.5**	**1.3–1.7**
Lifetime number of sex partners				
0–1 (Ref.)				
2–3	**5.2**	**1.6–17**	**1.8**	**1.5–2.1**
≥4	**8.6**	**2.8–26**	**2.9**	**2.4–3.6**
Unknown/Missing	**13**	**2.4–71**	1.3	0.91–2.0
Circumcision status (males only)				
Circumcised (Ref.)				
Not circumcised	2.4	0.49–11	**4.3**	**3.4–5.4**
Ever tested for HIV				
Yes (Ref.)				
No	**4.1**	**1.5–11**	**0.33**	**0.26–0.42**
Genital ulcer/sore in last 12 months				
Yes	0.91	0.25–3.3	**1.8**	**1.4–2.3**
No (Ref.)				
Used condom at last sex in last 12 months				
Yes (Ref.)				
No	2.1	0.48–9.7	**0.15**	**0.13–0.18**
Not sexually active	0.56	0.10–3.1	**0.44**	**0.37–0.53**
Unknown/Missing	1.9	0.36–10	**0.38**	**0.28–0.52**

Recent and long-term HIV infection according to full RITA based on LAg-avidity assay, antiretroviral treatment (self-report and biomarker) and viral load ≥1,000 c/mL. Items in bold are significant (CI excludes 1.0). Odds ratios rounded to two significant digits.

We also assessed whether alternative models would result in different conclusions given the rarity of the recent outcome, of which there were only 33 cases in 2012 and 2018 combined. We concluded that the use of standard maximum likelihood estimation does not appear to cause significant inflation of estimates compared with the Firth penalized likelihood estimation method, which produces unbiased estimates (Supplementary Appendix SA1 and Supplementary Table S4).

Female sex (aOR = 3.1, 95% CI 2.6–3.7), residing in former Nyanza or Western Provinces (aOR = 2.3, 95% CI 1.9–2.6) versus elsewhere, males being uncircumcised versus circumcised (aOR = 4.3, 95% CI 3.4–5.4), having 2–3 lifetime sex partners (aOR = 1.8, 95% CI 1.5–2.1) or ≥4 partners (aOR = 2.9, 95% CI 2.4–3.6) and having a genital ulcer/sore in last 12 months (aOR = 1.8, 95% CI 1.4–2.3) were at increased odds of long-term HIV infection versus no infection.

In addition, those aged 15–24 (aOR = 0.17, 95% CI 0.13–0.20) or 25–34 (aOR = 0.64, 95% CI 0.56–0.74) versus 35–64 years old, those not tested for HIV in last 12 months (aOR = 0.33, 95% CI 0.26–0.42) and those who did not use a condom at last sex (aOR = 0.15, 95% CI 0.13–0.18) or were not sexually active (aOR = 0.44, 95% CI 0.37–0.53) versus those who did use a condom at last sex in last 12 months were at reduced odds of long-term HIV infection versus uninfected status.

## Discussion

To our knowledge, this is the first study to compare direct biomarker-based estimates of recent HIV infection from three nationally representative surveys. We found an HIV incidence of 0.14% (95% CI 0.057–0.23) per year among inhabitants aged 15–64 in 2018, with significant reductions in the percentage of HIV infections that were recent from 2007 to 2018, consistent with a decline in national HIV incidence in Kenya over the period. Both of these findings were consistent with recent UNAIDS adult 15–49 years incidence projections of a 66% drop from 0.37% to 0.13% from 2007 to 2018.^1^ However, the reduction from 2007 to 2012 was not significant, while the reduction from 2012 to 2018 was significant overall, for females, and adults aged 25–49 years.

These reductions in recent HIV infections are likely due to multiple factors, including the scale-up of biomedical prevention interventions, including voluntary medical male circumcision (VMMC) in traditionally noncircumcising communities,^26^ expansion of ART eligibility in 2012 and 2016, and rapid increases in HIV testing, diagnosis, and treatment leading to reduced population HIV viral load and subsequent transmission. Although disparities in educational attainment and household wealth were not consistently linked to greater individual risk of recent HIV infection as shown in Rakai, Uganda,^27^ societal changes, such as increasing household wealth and increasing educational attainment, as well as behavioral changes may have contributed to overall reductions in HIV incidence.^28^

With only 33 recent infections identified in the 2012 and 2018 surveys combined using the full RITA, there was limited power to explore factors associated with recent infection versus uninfected status. However, age 25–34 versus 35–64 years, having two or more lifetime sex partners, and never having tested for HIV were all independently associated with having a recent HIV infection. Factors that are typically associated with prevalent HIV infection^29^ were found in this analysis to also be associated with long-term HIV infection, including older age, female sex, lack of male circumcision, increasing number of lifetime sex partners, increased condom use (presumably influenced by knowledge of HIV-positive status), and self-reported STIs in the past year (acknowledging that a recent STI could not have caused a prior HIV infection).

Compared with Nairobi, the Nyanza region, in western Kenya, was associated with greater odds of long-term versus noninfection, while the Central region was associated with reduced odds of long-term versus noninfection.

Although it is encouraging to see that ever having an HIV test among those recently infected increased from 2012 to 2018, to reduce the risk of onward transmission, it is important to quickly identify new HIV infections. Continued expansion of strategies to identify those at greater risk of infection, such as testing of partners of HIV-infected index cases, is needed. As HIV incidence continues to decrease in many settings, there will be increasing feasibility and value to testing everyone newly diagnosed with HIV for recent HIV infection, enabling a better understanding of risk-factors of acquiring HIV-1 infection and improving the targeting of prevention to accelerate epidemic containment. Surveillance of recent HIV infections among all newly diagnosed with HIV is now being implemented in several countries, including in Kenya,^30^ and has the additional benefit of being scalable to key populations, which is typically not available through household surveys.

This study had several limitations. While we took steps to improve their comparability, each survey was implemented under a separate protocol, with differences in survey procedures and HIV testing algorithm. Although the procedure for ensuring participants knew their HIV diagnosis differed between surveys, all three surveys had similar response rates, with 88%, 85%, and 91% of interviewees aged 15–64 years agreeing to provide a specimen for HIV testing in 2007, 2012, and 2018, respectively.^2,20,21^ The use of a harmonized RITA, while necessary, resulted in higher point estimates for percentage recent than obtained from the full RITA, perhaps due to differential classification of elite controllers.^31^

The RITA outcomes were unavailable for 2.5% of eligible participants in 2007 and 2012. Regional or temporal HIV subtype heterogeneity could have played a role in the observed variation of recent infection.^19,32,33^ This study also had several strengths as the following: the use of repeat cross-sectional survey estimates ensured representativeness of the target population and required no assumptions about intervention effectiveness, unlike cohort and model-based incidence estimates, respectively.

The decreasing trend in recent versus long-term HIV infection, especially from 2012 to 2018, bodes well for the eventual control of HIV as a public health challenge in Kenya. Achieving a ratio of new HIV infections to deaths among people living with HIV less than one (with high ART coverage) has been proposed as a metric for epidemic transition,^16,34,35^ as it will result in a decreasing population of people living with HIV over time. This ratio was 1.11 (0.71–1.82) in Kenya in 2020,^36^ suggesting that declines in HIV incidence must accelerate to eclipse decreases in mortality brought about by improved access to ART.

However, achieving such a reduction in HIV incidence may hinge on implementing the evidence-based strategies for HIV prevention and public health surveillance defined in the latest Kenya AIDS Strategic Framework,^37^ including improved STI treatment, condom programming, continuing expansion of Pre-exposure Prophylaxis (PrEP) and VMMC, and recent HIV infection surveillance.

## Data Sharing Statement

The KAIS 2007 and KAIS 2012 deidentified survey datasets and accompanying metadata are available upon request from the National AIDS & STI Control Programme (NASCOP), Ministry of Health, Kenya, by contacting head@nascop.or.ke or for download from the Kenya National Bureau of Statistics National Data Archive at https://statistics.knbs.or.ke after electronic registration and agreement to terms of use. Once the final report is published, the KENPHIA 2018 deidentified datasets and metadata will be available from NASCOP by contacting head@nascop.or.ke, or for download from ICAP https://phia.icap.columbia.edu/data after electronic registration and agreement to terms of use. Use of the datasets is subject to restrictions.

## Supplementary Material

Supplemental data

Supplemental data

Supplemental data

Supplemental data

Supplemental data

Supplemental data

## Supplementary Material

Supplementary Table S4

Supplementary Appendix SA1
