# Supplementary material for: HIV Incidence, Recent HIV Infection, and Associated Factors, Kenya, 2007–2018
Source: AIDS Res Hum Retroviruses. 2023 Feb 8;39(2):57–67. doi: 10.1089/aid.2022.0054 (PMC9942172; doi:10.1089/aid.2022.0054)
Supplement: Supplemental data [file Suppl_TableS1.docx]

**Table S1. Detailed biomarker results for LAg-recent respondents, Kenya, 2007-18**

| 2007 |  | ARV biomarker | | | |
| --- | --- | --- | --- | --- | --- |
|  |  | Detected | Not detected | Missing | Total |
| Self-reported ART status | On ART | 5 | 0 | 4 | 9 |
|  | Not on ART | 4 | **41** | **22** | 67 |
|  | Missing | 0 | **0** | 0 | 0 |
|  | Total | 9 | 41 | 26 | 76 |

Note: total ‘not on ART’ for purposes of harmonized RITA = 63.

| 2012 |  | ARV biomarker | | | |
| --- | --- | --- | --- | --- | --- |
|  |  | Detected | Not detected | Missing | Total |
| Self-reported ART status | On ART | 31 | 1 | 5 | 37 |
|  | Not on ART | 6 | **23** | **6** | 35 |
|  | Missing | 0 | **0** | 0 | 0 |
|  | Total | 37 | 24 | 11 | 72 |

Note: total ‘not on ART’ for purposes of harmonized RITA = 29.

| 2018 |  | ARV biomarker | | | |
| --- | --- | --- | --- | --- | --- |
|  |  | Detected | Not detected | Missing | Total |
| Self-reported ART status | On ART | 127 | 8 | 0 | 135 |
|  | Not on ART | 18 | **20** | **0** | 38 |
|  | Missing | 2 | **0** | 0 | 2 |
|  | Total | 147 | 28 | 0 | 175 |

Notes: LAg, limiting antigen (LAg)-avidity assay; RITA = recent infection testing algorithm; ARV = antiretroviral. Total ‘not on ART’ for purposes of harmonized RITA = 20. Those considered ‘not on ART’ for purposes of RITA are shown in bold / grayed cells.
