# Supplementary material for: HIV Incidence, Recent HIV Infection, and Associated Factors, Kenya, 2007–2018
Source: AIDS Res Hum Retroviruses. 2023 Feb 8;39(2):57–67. doi: 10.1089/aid.2022.0054 (PMC9942172; doi:10.1089/aid.2022.0054)
Supplement: Supplemental data [file Suppl_TableS2.docx]

**Table S2. Percentage of recent HIV infections among HIV positives by year and by recent infection testing algorithm, using original KENPHIA weights and including North Eastern, Kenya, 2007–18**

|  | **Percentage of those HIV-positive with a recent infection** | |  | **Change in percentage of recent infections*** | | |
| --- | --- | --- | --- | --- | --- | --- |
|  |  |  |  | **Harmonized RITA** | | **Full RITA** |
| **Year** | **Harmonized RITA**  **n/N^ⴕ^ (%; 95% CI)** | **Full RITA**  **n/N^ⴕ^ (%; 95% CI)** |  | **Vs. 2007**  **aOR**  **(p-value)** | **Vs. 2012**  **aOR**  **(p-value)** | **Vs. 2012**  **aOR**  **(p-value)** |
| **2007** | 64/1,024  (6·3; 4·2–8·4) |  |  |  |  |  |
| **2012** | 29/569  (5·6; 3·0–8·2) | 22/569  (4·1; 1·7–6·5) |  | 0·95 (0·87) |  |  |
| **2018** | 20/1,523  (1·7; 1·2–2·2) | 11/1,523  (0·95; 0·67–1·2) |  | **0·29 (<0·001)** | **0·31 (0·0014)** | **0·25 (0·0033)** |

Notes: * controlling for sex and age group. ⴕ n are unweighted. Harmonized recent HIV infection testing algorithm (RITA) is based on limiting antigen (LAg)-avidity and antiretroviral treatment (self-report and biomarker), full RITA is based on LAg-avidity, antiretroviral treatment (self-report and biomarker) and viral load ≥1,000 copies/milliliter. All estimates adjusted for survey designs. Items in bold are significant (p<0·05). Percentages, p-values and odds ratios rounded to two significant digits.
