# Supplementary material for: HIV Incidence, Recent HIV Infection, and Associated Factors, Kenya, 2007–2018
Source: AIDS Res Hum Retroviruses. 2023 Feb 8;39(2):57–67. doi: 10.1089/aid.2022.0054 (PMC9942172; doi:10.1089/aid.2022.0054)
Supplement: Supplemental data [file Suppl_FigS1.docx]

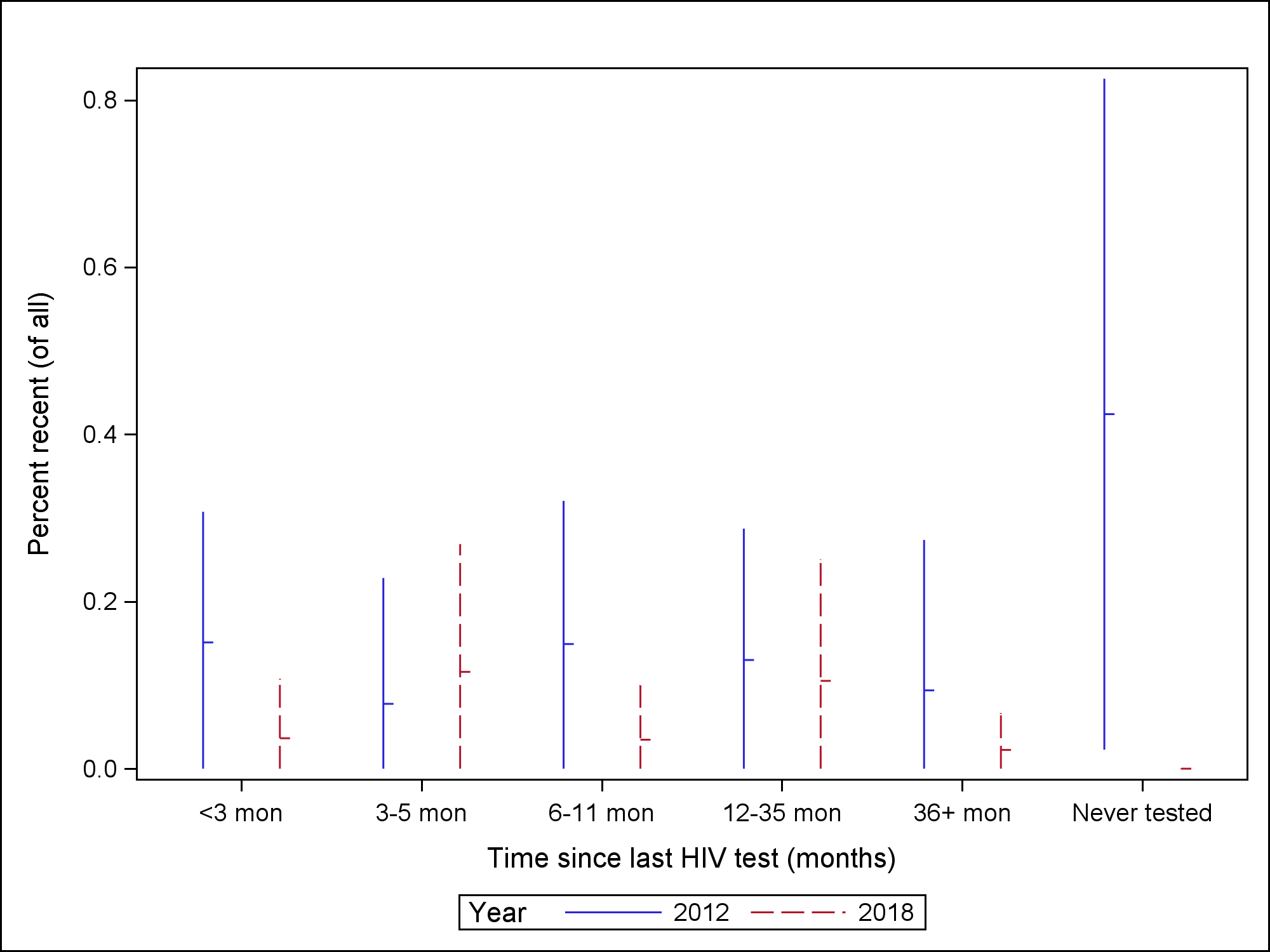


**Figure S1.** Percent recent (among all HIV-infected and not infected) versus history of HIV testing, by survey year, Kenya, 2012 and 2018**.** Point estimates and 95% confidence intervals shown.
