# Supplementary material for: HIV Incidence, Recent HIV Infection, and Associated Factors, Kenya, 2007–2018
Source: AIDS Res Hum Retroviruses. 2023 Feb 8;39(2):57–67. doi: 10.1089/aid.2022.0054 (PMC9942172; doi:10.1089/aid.2022.0054)
Supplement: Supplemental data [file Suppl_TableS3.docx]

**Table S3. Number recent, long-term and not HIV infected versus history of HIV testing, by survey year, Kenya 2012 and 2018**

|  | **2012** | | | **2018** | | |
| --- | --- | --- | --- | --- | --- | --- |
| **Time since last HIV test / HIV status** | **N** | **Col %** | **95% CI** | **N** | **Col %** | **95% CI** |
| < 3 months | 1,584 | 100 | (0–0) | 3,977 | 100 | (0–0) |
| Recent | 4 | 0·15 | (0–0.31) | 1 | 0·04 | (0–0.11) |
| Long-term | 121 | 7·8 | (6.3–9.4) | 307 | 6·9 | (5.9–7.9) |
| Uninfected | 1,459 | 92 | (90–94) | 3,669 | 93 | (92–94) |
| 3-5 months | 1,353 | 100 | (0–0) | 3,409 | 100 | (0–0) |
| Recent | 1 | 0·08 | (0–0.23) | 3 | 0·12 | (0–0.27) |
| Long-term | 74 | 5·9 | (4.3–7.4) | 185 | 5·2 | (4.2–6.2) |
| Uninfected | 1,278 | 94 | (92–96) | 3,221 | 95 | (94–96) |
| 6-11 months | 1,618 | 100 | (0–0) | 3,469 | 100 | (0–0) |
| Recent | 3 | 0·15 | (0–0.32) | 2 | 0·03 | (0–0.100) |
| Long-term | 66 | 4·4 | (3.1–5.6) | 144 | 3·9 | (3.1–4.7) |
| Uninfected | 1,549 | 95 | (94–97) | 3,323 | 96 | (95–97) |
| 12-35 months | 2,210 | 100 | (0–0) | 4,829 | 100 | (0–0) |
| Recent | 4 | 0·13 | (0–0.29) | 2 | 0·11 | (0–0.25) |
| Long-term | 99 | 4·4 | (3.4–5.4) | 264 | 4·9 | (4.2–5.6) |
| Uninfected | 2,107 | 95 | (94–96) | 4,563 | 95 | (94–96) |
| 36+ months | 1,487 | 100 | (0–0) | 2,982 | 100 | (0–0) |
| Recent | 2 | 0·09 | (0–0.27) | 1 | 0·02 | (0–0.066) |
| Long-term | 122 | 8·4 | (6.6–10) | 402 | 13 | (12–15) |
| Uninfected | 1,363 | 91 | (90–93) | 2,579 | 87 | (85–88) |
| Never tested | 3,191 | 100 | (0–0) | 5,492 | 100 | (0–0) |
| Recent | 8 | 0·42 | (0.023–0.83) | 0 | ·· | (0–0) |
| Long-term | 54 | 1·7 | (1.2–2.2) | 78 | 1·3 | (0.89–1.7) |
| Uninfected | 3,129 | 98 | (97–98) | 5,414 | 99 | (98–99) |
| Missing | 104 | 100 | (0–0) | 2,296 | 100 | (0–0) |
| Recent | 0 | ·· | (0–0) | 2 | 0·05 | (0–0.12) |
| Long-term | 11 | 10 | (4.5–16) | 130 | 5·2 | (4.0–6.3) |
| Uninfected | 93 | 90 | (84–96) | 2,164 | 95 | (94–96) |
| Total | 11,547 | 100 | (0–0) | 26,454 | 100 | (0–0) |
| Recent | 22 | 0·21 | (0.083–0.33) | 11 | 0·05 | (0.015–0.087) |
| Long-term | 547 | 4·8 | (4.2–5.5) | 1,510 | 5·3 | (4.9–5.7) |
| Uninfected | 10,978 | 95 | (94–96) | 24,933 | 95 | (94–95) |

**Note:** Percentages rounded to two significant digits.
