# Supplementary material for: HIV Incidence, Recent HIV Infection, and Associated Factors, Kenya, 2007–2018
Source: AIDS Res Hum Retroviruses. 2023 Feb 8;39(2):57–67. doi: 10.1089/aid.2022.0054 (PMC9942172; doi:10.1089/aid.2022.0054)
Supplement: Supplemental data [file Suppl_TableS4.docx]

**Table S4. Sensitivity analysis for multiple logistic regression for recent infection, Kenya, 2012-18**

|  | Recent infection compared to HIV-uninfected | | | | | | Relative difference in methods (%) | | | |
| --- | --- | --- | --- | --- | --- | --- | --- | --- | --- | --- |
| Characteristic | Multinomial, adjusted* | | Logistic, unadjusted+ | | Weighted, Firth regression^ | | Logistic, unadjusted | | Firth vs logistic, unadjusted | |
|  | aOR | (95% CI) | aOR | (95% CI) | aOR | (95% CI) | aOR (%) | CI (%) | aOR (%) | CI (%) |
| Year |  |  |  |  |  |  |  |  |  |  |
| 2012 | **3.16** | **(1.17–8.53)** | **3.17** | **(1.53–7.34)** | **3.01** | **(1.48–6.84)** | 0.164 | -21.1 | -4.91 | -7.76 |
| 2018 (ref) |  |  |  |  |  |  |  |  |  |  |
| Sex |  |  |  |  |  |  |  |  |  |  |
| Male (ref) |  |  |  |  |  |  |  |  |  |  |
| Female | 2.40 | (0.766–7.53) | **2.44** | **(1.20–5.00)** | **2.42** | **(1.20–4.93)** | 1.48 | -43.8 | 0.782 | -1.84 |
| Province |  |  |  |  |  |  |  |  |  |  |
| Nyanza/Western | 1.90 | (0.757–4.75) | **1.93** | **(1.04–3.50)** | **1.95** | **(1.06–3.50)** | 1.79 | -38.3 | 0.746 | 0.554 |
| Other (ref) |  |  |  |  |  |  |  |  |  |  |
| Age group (Years) |  |  |  |  |  |  |  |  |  |  |
| 15–24 | 3.13 | (0.758–12.9) | **3.23** | **(1.37–7.68)** | **3.20** | **(1.38–7.48)** | 3.27 | -48.1 | 0.926 | -3.39 |
| 25–34 | 4.24 | (1.39–12.9) | **4.36** | **(2.14–9.44)** | **4.25** | **(2.11–9.05)** | 3.00 | -36.6 | -2.62 | -4.89 |
| 35–64 (ref) |  |  |  |  |  |  |  |  |  |  |
| Education |  |  |  |  |  |  |  |  |  |  |
| No education / Primary (ref) |  |  |  |  |  |  |  |  |  |  |
| ≥Primary | 2.82 | (0.876–9.05) | **2.82** | **(1.31–7.08)** | **2.66** | **(1.26–6.47)** | 0.055 | -29.4 | -5.63 | -9.65 |
| Lifetime number of sex partners |  |  |  |  |  |  |  |  |  |  |
| 0–1 (ref) |  |  |  |  |  |  |  |  |  |  |
| 2–3 | **5.16** | **(1.59–16.8)** | **5.21** | **(1.91–17.7)** | **4.90** | **(1.85–15.7)** | 1.02 | 3.73 | -5.92 | -11.9 |
| ≥4 | **8.58** | **(2.80–26.3)** | **8.77** | **(3.07–30.7)** | **8.23** | **(2.95–27.4)** | 2.22 | 17.5 | -6.13 | -11.7 |
| Unknown/Missing | **13.2** | **(2.43–71.3)** | **13.1** | **(1.09–137.7)** | **11.7** | **(1.15–135.1)** | 0.67 | 98.4 | -10.9 | -1.95 |
| Circumcision status (males only) |  |  |  |  |  |  |  |  |  |  |
| Circumcised (ref) |  |  |  |  |  |  |  |  |  |  |
| Not circumcised | 2.37 | (0.492–11.4) | 2.50 | (0.661–7.23) | 2.73 | (0.787–7.55) | 5.53 | -39.7 | 9.34 | 3.04 |
| Ever tested for HIV |  |  |  |  |  |  |  |  |  |  |
| Yes (ref) |  |  |  |  |  |  |  |  |  |  |
| No | **4.09** | **(1.51–11.1)** | **4.14** | **(2.15–7.90)** | **4.13** | **(2.16–7.85)** | 1.13 | -39.9 | 0.123 | -1.11 |
| Genital ulcer/sore in last 12 mn |  |  |  |  |  |  |  |  |  |  |
| Yes | 0.909 | (0.251–3.29) | 0.920 | (0.132–3.19) | 1.15 | (0.218–3.59) | 1.25 | 0.647 | 25.4 | 10.2 |
| No (ref) |  |  |  |  |  |  |  |  |  |  |
| Used condom at last sex in last 12 mn |  |  |  |  |  |  |  |  |  |  |
| Yes (ref) |  |  |  |  |  |  |  |  |  |  |
| No | 2.15 | (0.475–9.72) | 2.23 | (0.804–8.74) | 1.96 | (0.745–6.94) | 3.63 | -14.2 | -12.0 | -21.9 |
| Not sexually active | 0.561 | (0.100–3.13) | 0.57 | (0.112–2.97) | 0.577 | (0.125–2.68) | 1.73 | -5.81 | 1.15 | -10.4 |
| Unknown/Missing | 1.90 | (0.361–10.0) | 1.96 | (0.161–16.3) | 2.25 | (0.168–16.2) | 3.17 | 67.1 | 14.3 | 0.735 |
| Median % difference (absolute value) |  |  |  |  |  |  | 1.73^β^ | -21.1 | 5.63^β^ | 3.39 |

Notes: *Multinomial, adjusted is multinomial logistic regression including survey design, implemented with PROC SURVEYLOGISTIC in SAS. ^+^Logistic, unadjusted is binary logistic regression without survey design implemented with PROC LOGISTIC in SAS. ^Weighted Firth regression is Firth penalized maximum likelihood estimation of binary logistic regression implemented with PROC LOGISTIC in SAS. ^β^ Median of absolute value of percent difference shown. Mn = months. aOR = adjusted odds ratio. Characteristics in bold would be considered significant based on confidence interval not including null (aOR=1) value.
