# Supplementary material for: HIV Incidence, Recent HIV Infection, and Associated Factors, Kenya, 2007–2018
Source: AIDS Res Hum Retroviruses. 2023 Feb 8;39(2):57–67. doi: 10.1089/aid.2022.0054 (PMC9942172; doi:10.1089/aid.2022.0054)
Supplement: Supplemental data [file Suppl_AppSA1.docx]

**Appendix S1. Sensitivity analyses related to logistic regression methods**

To understand how the selection of statistical modeling methods may have impacted the multiple regression results, we explored alternative models. The multinomial regression model reported in the manuscript and shown in Table 4 was chosen because it allowed simultaneous comparison of the magnitude of associations of factors associated with both recent and long-term infection consistently within a single, unified regression model. Furthermore, there are implementations of multinomial regression models available in statistics packages such as SAS’s SURVEYLOGISTIC procedure using the link=GLOGIT option which fits a generalized linear model accommodating multiple response levels in the outcome while also appropriately handling the complex survey design. However, the maximum likelihood estimation of the logistic regression model is known to result in meaningful bias when one of the response categories has few responses due to study of a rare outcome, as is the case with recent infection in this analysis, which can result in inflated parameter estimates.^1^ Several approaches have been proposed to address this problem including conditional logistic regression, exact logistic regression, and Firth’s penalized likelihood estimation (MLE),^2^ the latter being implemented in the SAS LOGISTIC procedure using the /FIRTH option under model statement. However, these approaches cannot currently accommodate the multinomial response categories we chose to model, nor to our knowledge, have they been extended to accommodate the survey design for variance estimation. Therefore, to compare the impact of these different approaches, we ran the reference method (survey-adjusted MLE) on a binary outcome of recent versus HIV-uninfected, which is the comparison of concern regarding small sample bias. We then contrast that with a simple weighted logistic regression and a weighted Firth logistic regression using Firth’s penalized MLE (Table S4). We also assessed exact logistic regression on a model with fewer covariates, but results were very similar to the reference method and are not shown here.

Generally, switching from multinomial survey logistic regression to simple binary logistic regression results in different results, with some factors showing slightly increased odds ratios (such as year 2012 vs 2018, female versus male sex, and province of Nyanza or Western versus other). The confidence intervals are also narrower, as expected, with a median reduction in width of 21.2%. Introducing the Firth penalized MLE estimation and profile likelihood confidence intervals results in small changes compared to the binary logistic point estimates – a median decrease of 5.63% with a range of -12.0% to 25.4%. The profile likelihood confidence intervals are slightly narrower than the Wald intervals with a 3.39% median decrease in width, range -21.9 to 10.2%. While there is evidence of modest bias in adjusted odds ratios due to the small sample size, the reduced width of the confidence intervals due to removal of the survey design from their estimation, along with the inability to model a multinomial response variable, would appear to outweigh the benefits of inclusion of the Firth penalized regression for fitting the regression algorithm, in this specific application.

**References:**

1. King G, Zeng L. Logistic regression in rare events data. J Stat Softw 2003;8:137–163; doi: 10.18637/jss.v008.i02.

2. Firth D. Bias reduction of maximum likelihood estimates. Biometrika 1993;80(1):27–38; doi: 10.1093/biomet/80.1.27.

**Table S4. Sensitivity analysis for multiple logistic regression for recent infection, Kenya, 2012-18**

|  | Recent infection compared to HIV-uninfected | | | | | | Relative difference in methods (%) | | | |
| --- | --- | --- | --- | --- | --- | --- | --- | --- | --- | --- |
| Characteristic | Multinomial, adjusted* | | Logistic, unadjusted+ | | Weighted, Firth regression^ | | Logistic, unadjusted | | Firth vs logistic, unadjusted | |
|  | aOR | (95% CI) | aOR | (95% CI) | aOR | (95% CI) | aOR (%) | CI (%) | aOR (%) | CI (%) |
| Year |  |  |  |  |  |  |  |  |  |  |
| 2012 | **3.16** | **(1.17–8.53)** | **3.17** | **(1.53–7.34)** | **3.01** | **(1.48–6.84)** | 0.164 | -21.1 | -4.91 | -7.76 |
| 2018 (ref) |  |  |  |  |  |  |  |  |  |  |
| Sex |  |  |  |  |  |  |  |  |  |  |
| Male (ref) |  |  |  |  |  |  |  |  |  |  |
| Female | 2.40 | (0.766–7.53) | **2.44** | **(1.20–5.00)** | **2.42** | **(1.20–4.93)** | 1.48 | -43.8 | 0.782 | -1.84 |
| Province |  |  |  |  |  |  |  |  |  |  |
| Nyanza/Western | 1.90 | (0.757–4.75) | **1.93** | **(1.04–3.50)** | **1.95** | **(1.06–3.50)** | 1.79 | -38.3 | 0.746 | 0.554 |
| Other (ref) |  |  |  |  |  |  |  |  |  |  |
| Age group (Years) |  |  |  |  |  |  |  |  |  |  |
| 15–24 | 3.13 | (0.758–12.9) | **3.23** | **(1.37–7.68)** | **3.20** | **(1.38–7.48)** | 3.27 | -48.1 | 0.926 | -3.39 |
| 25–34 | 4.24 | (1.39–12.9) | **4.36** | **(2.14–9.44)** | **4.25** | **(2.11–9.05)** | 3.00 | -36.6 | -2.62 | -4.89 |
| 35–64 (ref) |  |  |  |  |  |  |  |  |  |  |
| Education |  |  |  |  |  |  |  |  |  |  |
| No education / Primary (ref) |  |  |  |  |  |  |  |  |  |  |
| ≥Primary | 2.82 | (0.876–9.05) | **2.82** | **(1.31–7.08)** | **2.66** | **(1.26–6.47)** | 0.055 | -29.4 | -5.63 | -9.65 |
| Lifetime number of sex partners |  |  |  |  |  |  |  |  |  |  |
| 0–1 (ref) |  |  |  |  |  |  |  |  |  |  |
| 2–3 | **5.16** | **(1.59–16.8)** | **5.21** | **(1.91–17.7)** | **4.90** | **(1.85–15.7)** | 1.02 | 3.73 | -5.92 | -11.9 |
| ≥4 | **8.58** | **(2.80–26.3)** | **8.77** | **(3.07–30.7)** | **8.23** | **(2.95–27.4)** | 2.22 | 17.5 | -6.13 | -11.7 |
| Unknown/Missing | **13.2** | **(2.43–71.3)** | **13.1** | **(1.09–137.7)** | **11.7** | **(1.15–135.1)** | 0.67 | 98.4 | -10.9 | -1.95 |
| Circumcision status (males only) |  |  |  |  |  |  |  |  |  |  |
| Circumcised (ref) |  |  |  |  |  |  |  |  |  |  |
| Not circumcised | 2.37 | (0.492–11.4) | 2.50 | (0.661–7.23) | 2.73 | (0.787–7.55) | 5.53 | -39.7 | 9.34 | 3.04 |
| Ever tested for HIV |  |  |  |  |  |  |  |  |  |  |
| Yes (ref) |  |  |  |  |  |  |  |  |  |  |
| No | **4.09** | **(1.51–11.1)** | **4.14** | **(2.15–7.90)** | **4.13** | **(2.16–7.85)** | 1.13 | -39.9 | 0.123 | -1.11 |
| Genital ulcer/sore in last 12 mn |  |  |  |  |  |  |  |  |  |  |
| Yes | 0.909 | (0.251–3.29) | 0.920 | (0.132–3.19) | 1.15 | (0.218–3.59) | 1.25 | 0.647 | 25.4 | 10.2 |
| No (ref) |  |  |  |  |  |  |  |  |  |  |
| Used condom at last sex in last 12 mn |  |  |  |  |  |  |  |  |  |  |
| Yes (ref) |  |  |  |  |  |  |  |  |  |  |
| No | 2.15 | (0.475–9.72) | 2.23 | (0.804–8.74) | 1.96 | (0.745–6.94) | 3.63 | -14.2 | -12.0 | -21.9 |
| Not sexually active | 0.561 | (0.100–3.13) | 0.57 | (0.112–2.97) | 0.577 | (0.125–2.68) | 1.73 | -5.81 | 1.15 | -10.4 |
| Unknown/Missing | 1.90 | (0.361–10.0) | 1.96 | (0.161–16.3) | 2.25 | (0.168–16.2) | 3.17 | 67.1 | 14.3 | 0.735 |
| Median % difference (absolute value) |  |  |  |  |  |  | 1.73^β^ | -21.1 | 5.63^β^ | 3.39 |

Notes: *Multinomial, adjusted is multinomial logistic regression including survey design, implemented with PROC SURVEYLOGISTIC in SAS. ^+^Logistic, unadjusted is binary logistic regression without survey design implemented with PROC LOGISTIC in SAS. ^Weighted Firth regression is Firth penalized maximum likelihood estimation of binary logistic regression implemented with PROC LOGISTIC in SAS. ^β^ Median of absolute value of percent difference shown. Mn = months. aOR = adjusted odds ratio. Characteristics in bold would be considered significant based on confidence interval not including null (aOR=1) value.
